# Constraints on the Structure of the Shallow Crust in Central Italy from Geophysical Log Data

**DOI:** 10.1038/s41598-020-60855-0

**Published:** 2020-03-02

**Authors:** Paola Montone, Maria Teresa Mariucci

**Affiliations:** Istituto Nazionale di Geofisica e Vulcanologia, Via di Vigna Murata 605, 00143 Roma, Italy

**Keywords:** Geology, Geophysics, Seismology, Tectonics

## Abstract

To better define the seismic velocities of the shallow crust in central Italy, in the area affected by the 1997 Colfiorito, 2009 L’Aquila and 2016–2018 Amatrice–Norcia seismic sequences, we selected all deep wells with available sonic logs from the Apennine belt to the related Adriatic foredeep. Sonic logs are among the most important *in situ* measurements of rock properties and provide a reliable image of physical conditions at depth. By analysing the wave train transit times, we inferred the P-wave velocity within depth intervals displaying homogeneous sonic log properties, and estimated the rock density by applying an empirical relationship between the sonic velocity and density in sedimentary rocks. We compared these results with the main litho-stratigraphic units in stratigraphic profiles of the wells. From the density estimates, we inferred the trends of the vertical stress magnitude in the belt, eastern front and foredeep geodynamic domains. This work is a contribution to better interpretation of physical conditions at depth and provides data that can be applied to define more complete seismological, gravity and magnetic models. We provide data uncertainties that must be considered to ensure proper use of data and to evaluate the spatial resolution of the models derived from those data.

## Introduction

Information on P-wave velocity (Vp) data and the velocity model obtained from that information represent a first step, within the limits of geological and geophysical data, to constrain the tectonic structure of the crust in complex regions. In seismically active areas, a reliable velocity model contributes to the accuracy of hypocenter earthquake locations and focal mechanism solutions, and to define the main velocity discontinuities retrieved by, for example, tomographic models [e.g.^1–3^]. Sonic logs are the most significant measurements of *in situ* rock properties giving a reliable image of shallow crust conditions, despite their limits mainly due to the explored rock volume and the working frequency of the tool.

**Figure 1:**
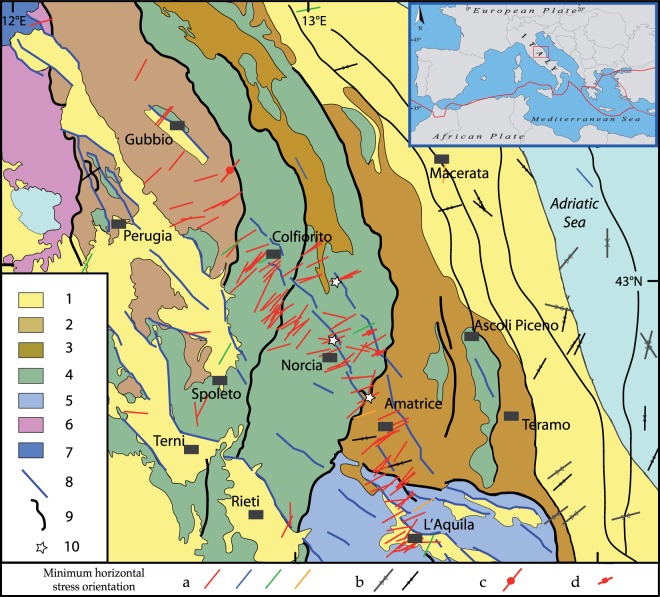
Geological and tectonic features of the study area with present-day stress data. Legend: (1) marine and continental Plio-Quaternary sediments; (2) Marnoso Arenacea Formation (Miocene); (3) Laga Formation (Miocene–Pliocene); (4) Carbonate sedimentary sequence of the Umbria–Marche domain (Mesozoic–Cenozoic); (5) Carbonate sedimentary sequence of the Latium–Abruzzo domain (Mesozoic–Cenozoic); (6) Tuscany nappe units; (7) Liguridi unit; (8) normal fault; (9) thrust fault; (10) Three mainshocks of the 2016 central Italy seismic sequence. Minimum horizontal stress orientations^22^ from: (**a**) earthquake focal mechanisms (red: normal; blue: reverse; green: strike-slip; orange: normal-strike); (**b**) borehole breakout data (the large grey symbol indicates better-quality data than the small black symbol); (**c**) formal inversion of earthquake focal mechanisms; (**d**) fault data. Tectonics and geological data are from^25,34^, and^49^. For detailed explanations of present-day stress indicators see^22^. The map has been generated with Esri ArcGIS Desktop 10.2 (www.esri.com).

Research to identify the P-wave velocities for the shallow crust was realised in central and northern Italy. In central Italy (Fig. 1) immediately after the Mw 6.0 24 August 2016 Amatrice earthquake^4,5^, additional borehole data on the stratigraphic profiles and sonic logs were obtained in the belt very close to the Amatrice area (two wells) and, to the east, along the Adriatic foredeep (two wells)^6^. In this part of Italy, the lithostatic gradient gradually changes from ~26 MPa/km close to Amatrice to less than 23 MPa/km along the foredeep. The overburden stress magnitude varies from 130 to 114 MPa at a depth of 5 km from the Apennine belt to the Adriatic foredeep, as a result of the differences in the P-wave velocities observed at the same depth in adjacent areas. At this depth, P-wave velocities of ~7 km/s in the belt and ~4 km/s along the foredeep have been measured.

In the northern Italy area including the Po Plain, the Apennine belt and Adriatic foredeep, a five-layer Vp model was gathered by the analysis of 64 deep wells; the overburden stress magnitude was calculated for each well, for the first time, in a wide area of Italy. Lithostatic gradients range from ~26 to 21 MPa/km from the belt through the Po Plain to the foredeep, with values of 130–105 MPa lithostatic pressure, at 5 km depth^7^.

Several crustal models have been proposed to explain the seismic velocity and density patterns at different scales and with different data coverage, all with the goal of characterising the crustal structure [i.e.^8–10^]. In particular, for the Italian peninsula, detailed information on the crustal and upper mantle structure has been obtained from regional high-resolution tomographic studies^11–14^ as well as from regional seismic reflection surveys^15^ and broadband waveform inversions^16^.

A recent paper provided a new tectonic interpretation of the central Apennine normal faulting system using P-wave velocity data from seismic profiles and deep wells^17^. Well-constrained P-wave data have been also used to identify the main acoustic reflectors to ensure that seismic reflection lines are interpreted correctly. Previously^3^, analysed sonic log data from six deep boreholes (5 km depth), compared those data to direct measurements of P- and S-wave velocities performed *in situ* and in the laboratory, and integrated those measurements with detailed data from geological surveys^18^. reconstructed the deep setting of the northern Apennine thrust belt by integrating surface structural–geological, subsurface seismic reflection profile, and well data, and provided detailed P-velocity and density values from the surface to 40 km depth.

The study area possesses a complex structural framework derived from the interaction between Miocene–Pliocene compressive structures (resulting from the emplacement of the Apennine belt) and Quaternary extensional faults [e.g.^19–21^]. The extensional stress regime (minimum horizontal stress) is NE-oriented (Fig. 1); this orientation is related to the NW–SE normal faults, and is mainly inferred from earthquake focal mechanisms and borehole breakouts^22,23^.

In 2016, central Italy was struck by an important seismic sequence that included three mainshocks^4,24–27^: Amatrice (Mw 6.0) on 24 August; Visso–Ussita (Mw 5.9) on 26 October; and Norcia (the largest, Mw 6.5) on 30 October 2016. The seismic sequence is characterised by shallow crustal earthquakes (5–15 km depth) and by a normal faulting regime, with a NE–SW extension, consistent with the present-day stress regime^28,29^. A very large number of papers have been produced after the seismic sequence, containing different seismotectonic interpretations and models [e.g.^25,30–36^]. However, in the field a surface normal faulting pattern striking N135–165, SW-dipping and subordinately NE-dipping, along ~28 km of the active Mt. Vettore–Mt. Bove fault system has been recognised, with a total surface rupture length of ~45 km^27,37–40^.

**Figure 2:**
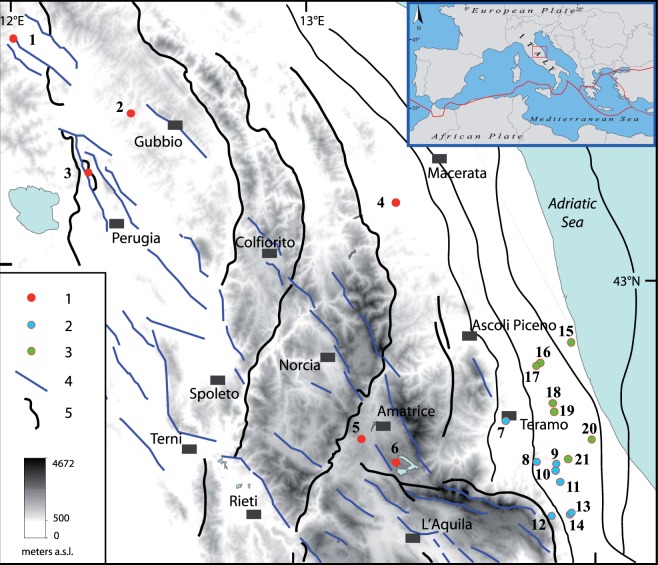
Locations of the deep boreholes analysed in this study: (1) inner Apennine belt group (wells from 445 to 1525 m above sea level); (2) eastern front group (wells from 183 to 684 m above sea level); (3) Adriatic foredeep group (wells from 33 to 391 m above sea level); (4) normal fault; (5) thrust fault. Wells 5, 6, 17 and 20 were previously analysed in^6^ and have been revised in the present paper. The map has been generated with Esri ArcGIS Desktop 10.2 (www.esri.com).

Following this destructive seismic sequence, we decided to conduct an in-depth study in the whole central Italy, because increased knowledge of the subsurface can markedly improve geophysical models. We selected 21 deep exploration wells for which sonic logs were available (Fig. 2), with locations in the inner Apennine belt, the eastern front, and the related Adriatic foredeep geodynamic domains. Data for four previously analysed wells^6^ are revised in the present paper.

**Figure 3:**
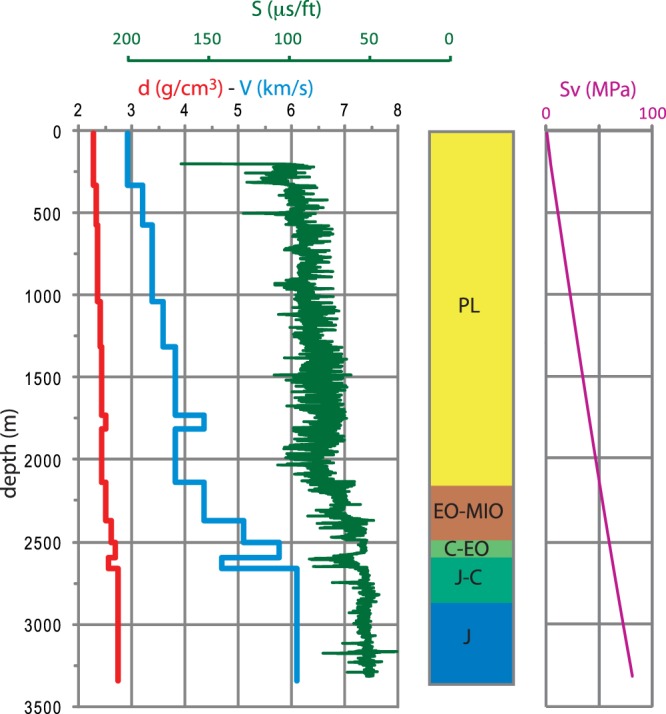
An example of the analysis. From the left: estimated density (red); inferred P-wave velocity (cyan); slowness raw data (green); litho-stratigraphic units (PL = Pliocene; EO–MIO = Eocene–Miocene; C–EO = Cretaceous–Eocene; J–C = Jurassic–Cretaceous; J = Jurassic); estimated vertical stress magnitude (Sv).

Sonic log data (Fig. 3) were used to improve characterisation of *in situ* P-wave velocities within depth intervals and velocity-derived density^41^ and compared with the stratigraphic profiles for both litho-stratigraphic units and each geological formation. Then, we calculated the vertical stress magnitudes for the three different domains from the surface to a maximum depth of ~7 km. Finally, we propose P-wave velocity and density mean values with their uncertainties for each litho-stratigraphic unit from the Quaternary deposits to the acoustic basement.

## Data and Results

**Figure 4:**
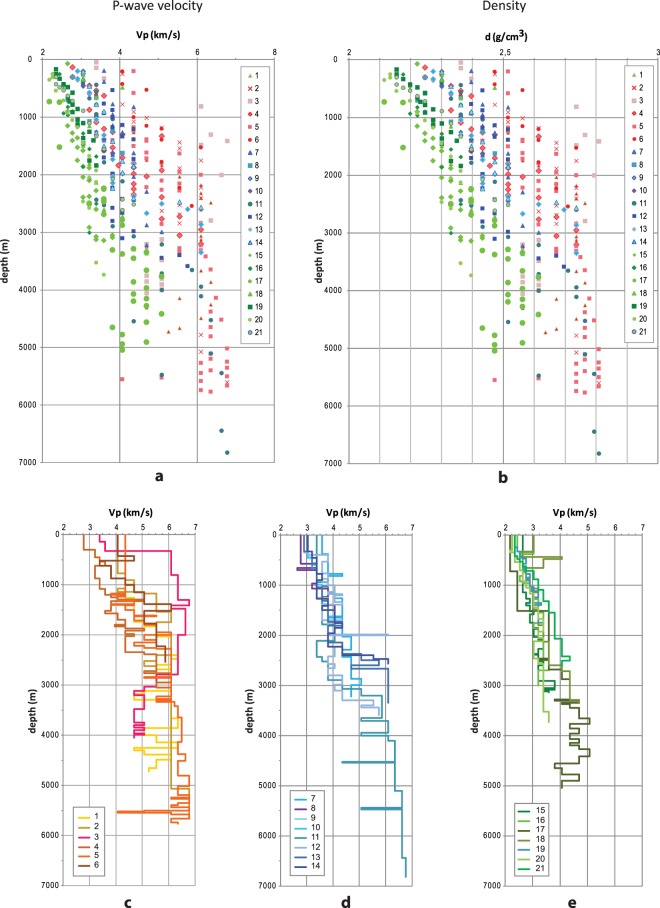
P-wave velocity (**a**) and density (**b**) trend with depth (below ground level) in the study wells. Values are plotted at the top depth. Red colours: Apennine belt area; blue colours: belt front area; green colours: Adriatic foredeep area. P-wave velocity along the wells in: (**c**) Apennine belt area (wells 1–6); (**d**) belt front area (wells 7–14); (**e**) Adriatic foredeep area (wells 15–21).

We analysed 21 sonic logs with a total length of more than 65 km (Supplementary information). The sampled depth ranges are between ~150 and 6800 m. The depth distribution of data, retrieved in each well as velocity or velocity-derived density, is plotted in Fig. 4(a,b). The wells are grouped on the basis of their geographic and structural position (the inner belt, the eastern front of the Apennine belt, and the Adriatic foredeep) to illustrate the different trends (Fig. 4c–e). All data exhibit large variability, probably resulting from different factors such as lithological complexity, the structural condition of the rock mass, and changes in the water content from a well to another, which cause the sonic response to vary over depths of hundred meter. The end members of the Vp distribution range from 2.2 km/s in the top hundred meters of the foredeep wells to 6.7 km/s in the inner belt wells, even at relatively shallow depths of around 1.5 km, probably resulting from variations in lithology. For example, in the belt wells at depths less than 0.5 km, the velocity can be about 2.7 or 6 km/s (Fig. 4c), whereas in the other groups the values are smaller but more similar for the same depth interval, between 2.8 and 3.8 km/s in the eastern front and from 2.2 to 3.0 km/s in the foredeep wells.

**Figure 5:**
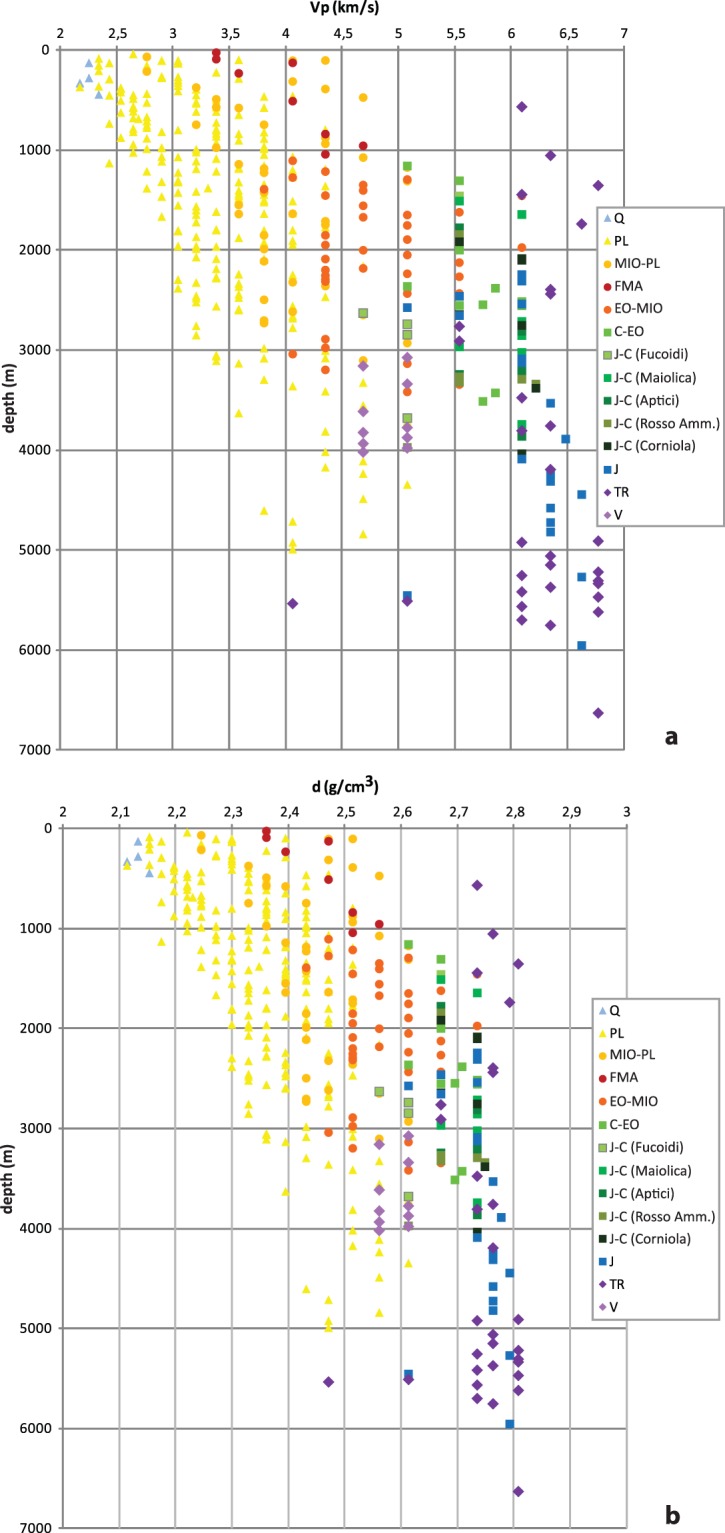
P-wave velocity (**a**) and density (**b**) values versus depth (below ground level) for litho-stratigraphic units or geological formations in the analysed wells. Values are plotted at the mean depth. Q, Quaternary (well 20); PL, Pliocene (wells 7–21); MIO–PL, Miocene–Pliocene (wells 4–7, 11, 12); FMA, Marnoso Arenacea (wells 2, 3); EO–MIO, Eocene–Miocene (wells 2, 4–6, 11–14); C–EO, Scaglia (wells 1, 2, 4–6, 11–14); J–C, Jurassic–Cretaceous formations (wells 1, 2, 4, 5, 11, 13): Fucoidi, Maiolica, Aptici, Rosso Ammonitico and Corniola formations; J, Jurassic (wells 1, 2, 4, 5, 11, 13); TR, Triassic (wells 1, 3–5, 11); V, Verrucano Formation s.l. (well 3). Detailed descriptions of the litho-stratigraphic units and geological formations are provided in the text.

A more useful comparative analysis of the whole dataset was performed comparing the sonic units with the stratigraphic units and deriving reference values for specific groups (Fig. 5). We simplified the well stratigraphy to group the units on the basis of the age of the geological formations, following also the RETRACE Project stratigraphic scheme^42^. The RETRACE-3D (centRal italy EarThquakes integRAted Crustal model) Project has been launched to build a new 3D model of the area. The project combines the multi-disciplinary skills of a large community of researchers and experts^42^, and provided us with data for seven wells (1, 3, 7, 8, 11, 13 and 18).

These litho-stratigraphic units include formations with similar mechanical properties: Quaternary (Q), Pliocene (PL), Miocene–Pliocene (MIO–PL), Marnoso Arenacea Formation (FMA), Eocene–Miocene (EO–MIO), Cretaceous–Eocene (C–EO), Jurassic–Cretaceous (J–C), Jurassic (J), Triassic (TR) and Verrucano Formation s.l. basement (V).

In this area, Q and PL are mainly marine clays and sands in different combinations and with different degrees of compaction and MIO–PL units include all the marly-arenaceous turbidite deposits (mainly Laga Formation). The FMA was considered separately and includes Neogene synorogenic deposits (turbidite succession). EO–MIO units consist of a marly–calcareous–siliceous succession at the top of the carbonate sequence. C–EO (Scaglia Formation) and J–C represent a largely carbonate sequence with a massive, shallow-water limestone (the Calcare Massiccio; J) at the base. TR denotes the Upper Triassic evaporitic sequence (Anidriti di Burano Formation) at the base of the carbonate sequence. Some minor groups are poorly represented in the study wells and it was not possible to compute significant mean values for these units; nevertheless, we provide values for the Permian to Middle Triassic clastic and metasedimentary rocks (phyllites) of the Verrucano Formation s.l. (V), which was present in only one well. Moreover, to assess the properties of specific stratigraphic intervals that often provide prominent seismic reflectors, we also analysed in detail the Jurassic–Cretaceous sequence. We split this sequence into its constituent geological formations (the Fucoidi, Maiolica, Aptici, Rosso Ammonitico and Corniola formations) to highlight any differences (Fig. 5a,b). Some unusual Vp values (with respect to the surroundings) were detected, for instance the low velocities of the Fucoidi (4.7 km/s), Rosso Ammonitico (5.1 km/s) and Aptici formations (5.5 km/s). The highest Vp values, 6.6 and 6.8 km/s, were detected in the Calcare Massiccio (J) and Anidriti di Burano (TR) formations, respectively. Some unexpectedly low values (about 4.1 and 5.1 km/s) are present in the Triassic units around 5.5 km depth; these values are apparently not related to compositional changes but probably to a fracture zone (Fig. 5a).

**Figure 6:**
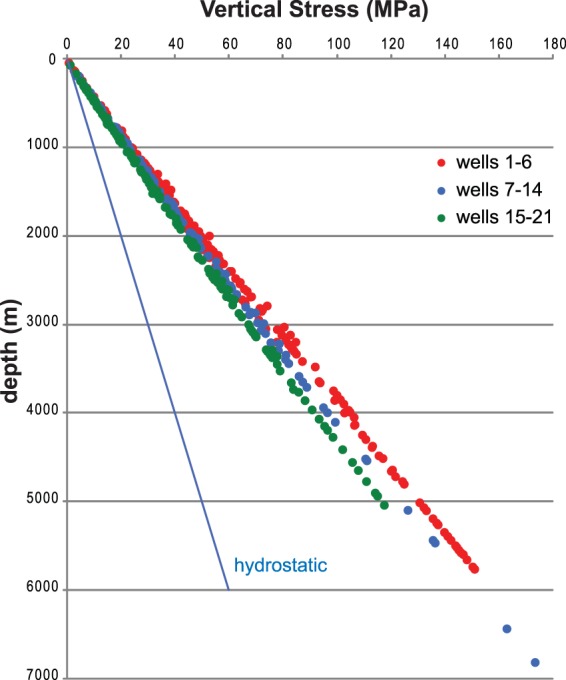
Vertical stress magnitude computed in each well. Depth below ground level. The blue line is the hydrostatic pressure. Red: wells in the Apennine belt area; blue: belt front area; green: Adriatic foredeep area.

Depth has a slight influence on the parameters, but only in less competent lithologies, for instance in the Pliocene–Pleistocene clays and sands, in which compaction effects occur. Velocity-derived density values were used to calculate more realistic vertical stress magnitudes (Fig. 6). Plotting of all data for different depths in all the wells reveals the existence of different lithostatic gradients (Sv) increasing from ~22 to ~26 MPa/km from the foredeep (wells 15–21) to the belt (wells 1–6). At a single depth, for instance at 5 km depth, the Sv difference between the belt and the foredeep is more than 10 MPa. A maximum Sv value of ~175 MPa at 6800 m depth was inferred from this dataset for well 11, which is located along the eastern front of the Apennines.

**Figure 7:**
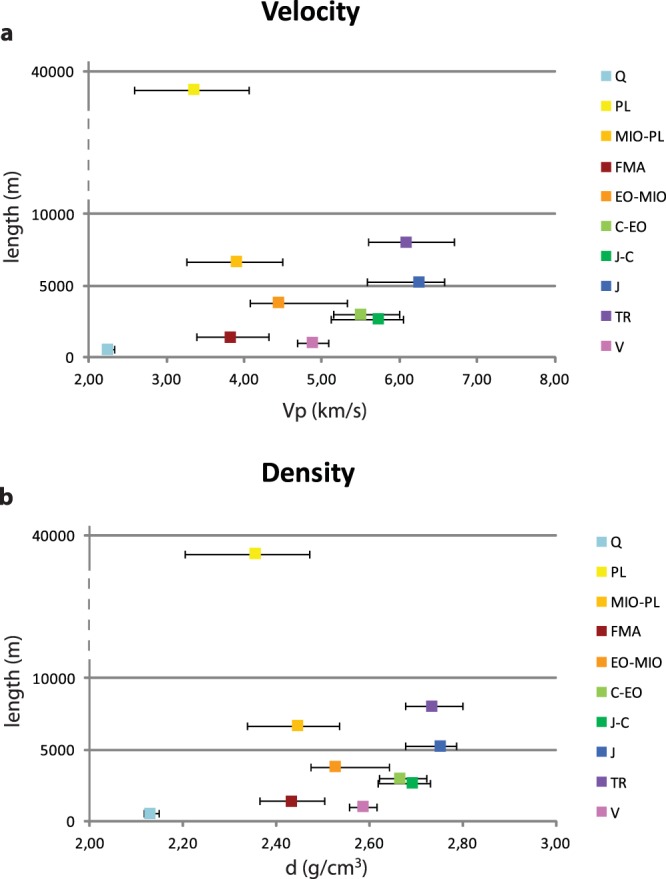
Mean value of the measurements versus the total thickness for each litho-stratigraphic unit. (**a**) P-wave velocity; (**b**) density. The mean value is the median (50^th^ percentile) and the error bars are the 10^th^ and 90^th^ percentiles of the cumulative distribution (FMA and V are the weighted mean).

We computed mean velocity and density values weighted by thickness for each stratigraphic unit as defined above. To describe the variability of the geophysical parameters, we calculate their cumulative distribution inside each stratigraphic unit; the median (50^th^ percentile) is the best estimation of the parameter and the 10^th^ to 90^th^ percentiles represent the natural variability (Fig. 7a,b). The stratigraphic units have different analysed total thickness: the Pliocene is the most represented and also the most variable as a result of the different depths and geomechanical characters of the considered units, for instance differences in the degree of consolidation, fracturing, porosity and water content. All the other units exhibit comparable analysed thicknesses, between approximately 1000 and 7000 m. The units with more variable lithology (e.g. EO–MIO, MIO–PL and PL) show a higher range of values, whereas those with consistent lithology (e.g. J, J-C and C–EO) have more restricted values even over a wide depth range (see also Fig. 5).

## Discussion

Knowledge of the physical *in situ* properties of rock sequences is of great importance to understand the structure of the earth’s crust. For this purpose, models were constructed using all the available geological and petrophysical constraints. We attempted to provide the most accurate *in situ* values by completely reviewing the available sonic data for central Italy taking to account the geologic and tectonic context.

Our results for P-wave velocity in the main litho-stratigraphic units compared to other studies are provided in Table 1. Given the uncertainties inherent in sonic records (Fig. 3, green curve), we provide differently by other papers, a range of errors for each P-velocity value. We report both the median value of the cumulative distribution and the 10^th^ to 90^th^ percentiles (Table 1, column 1) and the mean value weighted by thickness and standard deviation (Table 1, column 2). Our preferred computation is column 1 with added input from column 2: we used the data in column 2 when the cumulative distribution was not reliable, for instance when few data points or just one well were available. Columns 3 to 9 list the results obtained in previous studies^43^. considered a wide area when calculating velocity; for this reason, the differences between that study and other previous works are high. Finally, we also indicate any differences in the analysed litho-stratigraphic interval or any other necessary information (letters *a* to *g*).

**Table 1:** P-wave velocity (km/s) in the main units compared to other studies.

**This Study**			**Others**							
**Unit**	**1**	**2**	**3**- Porreca *et al*.		**4**- Latorre *et al*.	**5**- Scisciani *et al*.	**6**- Bigi *et al*.	**7**- Mirabella *et al*.	**8-** Barchi *et al*.	**9-** Bally *et al*.
Q	2.2 (2.2–2.3)	2.2 ± 0.1	Plio-Pleistocene			3.6		2.0 *(a)*	2.3–2.5	2.0
PL	3.4 (2.6–4.1)	3.4 ± 0.6								2.6
MIO-PL	4.0 (3.3–4.5)	4.0 ± 0.5	Miocene Turbidites	4.0		4.0	3.6 *(b)*			3.4–4.0 *(c)*
FMA		4.0 ± 0.3			3.9			4.0	4.0	
EO-MIO	4.4 (4.1–5.3)	4.8 ± 0.5	Marly group		5.7	5.0	4.5	5.6	4.0	3.4
C-EO	5.5 (5.1–6.0)	5.8 ± 0.3	Scaglia group	5.8			5.8		5.5	4.5
J-C	5.7 (5.1–6.0)	5.9 ± 0.4	Carbonate multilayer			5.5	6.1			5.2
J	6.3 (5.6–6.6)	6.3 ± 0.4	Calcare Massiccio Fm.			6.8	6.4			6.0
TR	6.1 (5.6–6.7)	6.3 ± 0.3	Evaporites	6.4	6.3	6.0	6.0	6.1	6.1	6.4
V		4.9 ± 0.2 *(d)*	Basement s.l. phyllites	5.1	5.3	5.5		5.1	5.0	3.9 *(e)*
			Crystalline basement unit		5.8 *(f)*	6.0		6.0 *(g)*	5.5	

Q, Quaternary; PL, Pliocene; MIO-PL, Miocene-Pliocene; FMA, Marnoso Arenacea Formation; EO-MIO, Eocene-Miocene; C-EO, Cretacic-Eocene; J-C, Jurassic-Cretacic; J, Jurassic; TR, Triassic; V, Verrucano s.l.; see text for a detailed description. 1- Median value of the cumulative distribution by length and 10^th^–90^th^ percentiles; 2- Mean weighted by length and standard deviation; 3- Porreca *et al*.^50^; 4- Latorre *et al*.^3^; 5- Scisciani *et al*.^18^; 6- Bigi *et al*.^51^; 7- Mirabella *et al*.^48^; 8- Barchi *et al*.^44^; 9- Bally *et al*.^43^. *(a)* Continental deposits; *(b)* Laga depositional sequence; *(c)* Messinian; *(d)* data from well n. 3 only; *(e)* data from “E-Adriatic offshore monocline”; *(f)* from^61^; *(g)* from^62^ and^63^.

During and after the late 1980s, results obtained from sonic or related log data from selected boreholes yielded significant information on the mean values of the seismic interval velocities. This information has mainly been used for depth conversion of seismic reflection profiles [e.g.^18,36,43–50^].

Our results in Table 1 and Fig. 7 are for the entire central Italy, nevertheless we wish to emphasise that: the borehole data for the Q and PL units are only from the eastern front and the Adriatic foredeep (15 wells); data for the MIO–PL (6 wells) and carbonate multilayer sequence (9 wells) were obtained from boreholes located along the inner belt and eastern front; the Triassic evaporite sequence is present in 4 belt boreholes and a well in the Apennine eastern front (well 11); the Verrucano basement was detected in a borehole in the inner belt (well 3).

In the following discussion, we do not report the associated errors for each single result so as not to overburden the reading. In the compilation of previously published P-wave velocity data for the lithologies in the central Italy area, the Plio-Pleistocene interval was characterised by values of 2–3.6 km/s; these values are consistent with our data for the Quaternary and the Pliocene intervals (Table 1). The results of the present study for the Miocene–Pliocene interval (MIO–PL) are in agreement with those of previous studies^50,51^ with values between 3.6 and 4.0 km/s.

We separately analysed the FMA interval (Marnoso Arenacea Formation, Miocene turbidites), although this unit is present in only two wells; in this case, we report the P-velocity mean weighted by thickness and standard deviation (Table 1, column 2). Our value is 4 km/s, which is quite similar to those reported by^3,48^, and^44^ for the same litho-stratigraphic interval. The underlying layer (EO–MIO) includes all the stratigraphic formations at the top of the carbonate sequence, and has an average Vp value of 4.4 km/s.

As shown in Table 1, some previous studies^3,48^ provided a single Vp value of 5.6–5.7 km/s for the J to EO–MIO succession, whereas other studies^44,50^ gave a value of 5.5–5.8 km/s for the sequence from J (massive limestone) to C–EO (carbonate layer). We prefer to consider each litho-stratigraphic unit separately, giving separate values for J, J–C and C–EO, as proposed by^18,51^, and^43^. Compared to previous studies^18,51^, our average results have slightly different values of 6.3, 5.7 and 5.5 km/s for J, J–C and C–EO, respectively. Another previous study obtained average Vp values of 5.5 and 6.5 km/s for carbonate rock and dolomite, respectively^17^.

Within the J–C litho-stratigraphic unit, we identified some characteristic low-velocity Vp markers: the Fucoidi Formation (Vp ~4.7 km/s); the Rosso Ammonitico Formation (Vp ~5.1 km/s) and the Aptici Formation (Vp ~5.5 km/s).

The Triassic evaporite Formation (TR) consists of a sequence of interbedded anhydrites and dolomites 1–2 km in thickness. Analysis in a previous study of the velocity frequency histograms of the sonic logs of three boreholes crossing Triassic evaporates (wells 1, 2 and 3) yielded most-registered velocities of 6.3 km/s for dolostones and 6.4 km/s for anhydrites^52^. Analysis of our data yielded an anhydrite P-velocity of 6.1 km/s for wells 1, 2 and 3 and values for dolostone of 6.4 km/s for well 1 and 6.8 km/s for wells 2 and 3. For the evaporites, the sonic log data are diagnostic in particular for dolostones, which have a constant, well-recognised velocity and transit time in the range 45 to 48 μs/ft, corresponding to a fast velocity value of up to 6.8 km/s; anhydrites instead exhibit a sonic measurement of 50 μs/ft, corresponding to a P-velocity of 6.1 km/s. Both rock types are present in the Triassic evaporite sequence (the Anidriti di Burano Formation); this unit has an average Vp value of 6.1 km/s (Table 1), indicating a prevalence of anhydrites within the formation.

In the investigated area, two deep wells penetrated the upper part of the acoustic basement (the Verrucano Formation); our data set includes one of these boreholes (well 3). Considering that the available sonic log data are for the upper portion of the Verrucano Formation (about 1000 m thickness), the obtained result (4.9 km/s) is in agreement with almost all previous data (Table 1).

The empirical relationship between density and velocity^41^ has been obtained from a large number of laboratory and field observations of different sedimentary rocks; however, the relationship cannot be applied to evaporitic rocks. To verify the obtained density values, we compared our velocity-derived density results with the bulk density log for well 2. Comparison for the depth interval from ~1000 to 2700 m, mainly for carbonate rocks, gives good results, although the velocity-derived densities are slightly higher by about 0.1 g/cm^3^. A very recent paper^36^ suggested density values for the basement, Triassic evaporites, calcareous rocks and turbidites of 2.55, 2.67, 2.48–2.58 and 2.25–2.40 g/cm^3^, respectively. Although within the range of error, these results are slightly lower than our estimates.

From the velocity-derived density data, we estimated the Sv magnitude as different values of lithostatic gradient for various geological areas and depths (Fig. 6). In the studied area, the lithostatic gradient changes from ~22 to ~26 MPa/km from the Adriatic foredeep to the inner belt. The maximum Sv value (~175 MPa) was measured at 6800 m depth in the central Apennine eastern front; thus, the values at the same depth in the inner belt may be higher.

The P-wave velocity is a function of the mechanical properties of a rock and is therefore proportional to the strength of the rock. As sediments become compacted, the elastic wave velocity linearly increases if the interval transit time, on a logarithmic scale, is plotted against depth. In our P-velocity data, which vary from a minimum value of 2.2 km/s to a maximum of 6.8 km/s in the topmost 7 km, this compaction trend is very evident within the clays and sands of the Pliocene group, whereas in the other litho-stratigraphic units it does not seem to be a typical feature (Fig. 5a).

In this work, we analysed only the sonic log data, although we are conscious that the variability of seismic wave velocity and density is a function also of temperature, pressure, chemical composition and water content^53,54^. Of all these factors, temperature may be the dominant influence on Vp and Vs, especially in areas of significant magmatism. Moreover, the sediment type may be relevant^54^, for instance in the Po Plain basin area (northern Italy) where sediments reach a thickness of 15 km^55^ or along the southern Adriatic foreland, which contains a 6–7-km-thick limestone sequence^56^.

In central Italy subsurface, P-wave velocity results account for important velocity lateral and at depth heterogeneities, highlighting the actual complexity of the area, now better defined by robust and reliable reference values. Within the three individual areas (inner belt, eastern front of the Apennines and Adriatic foredeep), the lateral P-wave velocity variation occurred mainly in the direction perpendicular to the Apennine structure. Only limitedly to the belt area, P-wave variation has been observed in the first 2 km depth (Fig. 4c), linked to the presence of different litho-stratigraphic units and to the complexity of the tectonic setting. At the same depth, this feature is not recognisable in the other two areas (Fig. 4d,e) where the litho-stratigraphic types are very similar (e.g. the Pliocene unit). Conversely, the high P-velocity difference in the eastern front is observable at deeper depth (Fig. 4d).

Similarly, for a section crossing the belt from the eastern front to the Adriatic foredeep, at the same depth P-wave variability is also apparent in the crustal volume by applying passive tomography techniques^26,57^. Using simplified velocity models results in considerable errors and systematic shifts in earthquake locations, mainly in tectonic contexts with important lateral variations and irregular topography^58^.

Geomechanical or geotechnical properties of rocks can be measured by static measurement of core specimens in the laboratory, or can be obtained using P-wave velocities from seismic, well log or laboratories-analysed core data. We analysed well logs with the future aim of constructing a more complete seismic velocity model to define earthquake locations and their correspondence with the subsurface geology and with tectonic structures more accurately.

The main goal of this paper is not to provide single better estimates, but estimates with their uncertainty. We wish to remark that the quantification of measurement errors is essential to check the presence of statistically significant variations of the seismic waves velocity, and to verify the sensitivity of the results of any model that uses such a kind of data.

Integration of *in situ* measurements of rock geomechanical properties is essential to improve velocity models for the crust and to better constrain the solutions of inversion procedures such as geophysical modelling. However, integration of this type of information must consider data uncertainties, both in terms of measurement errors and of the intrinsic natural variability within a litho-stratigraphic unit. In this paper we emphasise the role of these uncertainties to set a limit for the spatial resolution of the models that are derived from the data. If not properly considered, solutions of inversion procedures may contain small spatial details that are unrealistic because they are below the resolution of the data.

## Methods

Many deep wells have been drilled in Italy by oil companies. Although these wells are not homogeneously distributed, they are available as a contribution to estimating reliable petrophysical parameters of the shallow crust in different geodynamic contexts. Our analysis is mainly based on borehole sonic instrument recordings. This tool contains one or more transmitters that emit high-frequency acoustic waves, which travel through the rock formation to two or more receivers that record full waveforms. The difference in the arrival times of the sonic wave trains recorded by the detectors is used to determine the sonic velocity, despite the limits caused by the high frequency of the emissions, which are different from seismic waves, and to the small volume investigated around the borehole. The wave train transit times (slowness), usually in microseconds per foot, are generally between 40–140 μs/ft in sedimentary rocks.

We hand-picked slowness values with an accuracy/resolution of ±2 µs/ft to derive the P-wave velocity (Vp) through the formation (Fig. 3). First, we identified the “sonic units” in each well, that are intervals that correspond to zones of homogeneous values on the sonic log, and attributed a quality value to each interval. Then we compared these units to the stratigraphic log and other available geophysical logs (e.g. resistivity and gamma-ray data) to correlate between the different parameters.

We used the sonic velocity to predict density values, following the common practice in petrophysical investigations when density logs are not available (Fig. 3). To estimate density, we applied the empirical equation for sedimentary rocks^41^:

$${\rm{d}}=0.23\,{{\rm{V}}}^{0.25}$$

where d is density in g/cm^3^ and V is sonic velocity in ft/s.

Gardner’s relation^41^ is a good approximation for shales, sandstones and carbonates, although according to^59^, Gardner’s rule yields density values that are generally 0.1 g/cm^3^ or less higher than the Nafe–Drake curve^60^. Density estimations depart significantly from expected behaviour for evaporites. Average P-wave velocity and velocity-derived density values were computed for both the litho-stratigraphic units following the Retrace project and in the main formations of the Meso-Cenozoic sedimentary sequence of the study area (Fig. 5). We used the median value (50^th^ percentile) of the cumulative distribution within each group and the 10^th^ and 90^th^ percentiles as error bars (Fig. 7).

Density estimations were used to obtain more reliable vertical stress magnitude data at depth in each well and to assess the trend of the lithostatic gradient within the different sectors (Fig. 6).

## Supplementary information

Supplementary information.

## References

[1] Husen S (2003). Probabilistic earthquake location in complex three-dimensional velocity models: application to Switzerland. J. Geophys. Res..

[2] Wagner M, Husen S, Lomax A, Kissling E, Giardini D (2013). High-precision earthquake locations in Switzerland using regional secondary arrivals in a 3D velocity model. Geophys. J. Int..

[3] Latorre D, Mirabella F, Chiaraluce L, Trippetta F, Lomax A (2016). Assessment of earthquake locations in 3-D deterministic velocity models: a case study from the Altotiberina near fault observatory (Italy). J. Geophys. Res..

[4] http://terremoti.ingv.it.

[5] Tinti E, Scognamiglio L, Michelini A, Cocco M (2016). Slip heterogeneity and directivity of the ML 6.0 2016 Amatrice earthquake estimated with rapid finite-fault inversion. Geophys. Res. Lett..

[6] Mariucci, M. T. & Montone, P. Contemporary stress field in the area of the 2016 Amatrice seismic sequence (central Italy). *Ann. Geophys*. **59**(5), 10.4401/ag-7235 (2016).

[7] Montone P, Mariucci MT (2015). P-wave velocity, density, and vertical stress magnitude along the crustal Po Plain (Northern Italy) from sonic log drilling data. Pure Appl. Geophys..

[8] Molinari I, Morelli A (2011). EPcrust: a reference crustal model for the European Plate. Geophys. J. Int..

[9] Pasyanos ME, Masters TG, Laske G, Ma Z (2014). LITHO1. 0: an updated crust and lithospheric model of the Earth. J. Geophys. Res..

[10] Reguzzoni M, Sampietro D (2015). GEMMA: An Earth crustal model based on GOCE satellite data. Int. J. Appl. Earth Obs..

[11] Di Stefano R, Chiarabba C, Lucente F, Amato A (1999). Crustal and uppermost mantle structure in Italy from the inversion of P-wave arrival times: Geodynamic implications. Geophys. J. Int..

[12] Di Stefano R, Kissling E, Chiarabba C, Amato A, Giardini D (2009). Shallow subduction beneath Italy: three-dimensional images of the Adriatic-European-Tyrrhenian lithosphere system based on high-quality P wave arrival times. J. Geophys. Res..

[13] Brandmayr, E. *et al*. The lithosphere in Italy: structure and seismicity. In *The geology of Italy* (ed. Beltrando, M., Peccerillo, A., Mattei, M., Conticelli,S. & Doglioni C., Journal of the Virtual Explorer, Electronic edn, 36, paper 1 ISSN 1441-8142. http://virtual-explorer.com.au/article/2009/224/lithosphere-structure-seismicity (2010).

[14] Gualtieri L, Serretti P, Morelli A (2014). Finite-difference p wave travel time seismic tomography of the crust and uppermost mantle in the Italian region. Geochem. Geophys. Geosyst..

[15] Finetti, I. R. CROP project: deep seismic exploration of the Central Mediterranean and Italy in *Atlases of Geoscience*, vol. 1, 785 pp. (Elsevier, 2005).

[16] Herrmann RB, Malagnini L, Munafò I (2011). Regional moment tensors of the 2009 L’Aquila earthquake sequence. B. Seismol. Soc. Am..

[17] Buttinelli M., Pezzo G., Valoroso L., De Gori P., Chiarabba C. (2018). Tectonics Inversions, Fault Segmentation, and Triggering Mechanisms in the Central Apennines Normal Fault System: Insights From High‐Resolution Velocity Models. Tectonics.

[18] Scisciani V (2014). Positive inversion tectonics in foreland fold‐and thrust belts: a reappraisal of the Umbria–Marche northern Apennines (Central Italy) by integrating geological and geophysical data. Tectonophysics.

[19] Calamita F, Pizzi A (1994). Recent and active extensional tectonics in the southern Umbro-Marchean Apennines (central Italy). Mem. Soc. Geol. It..

[20] Galadini F, Galli P (2003). Paleoseismology of silent faults in the Central Apennines (Italy): the Mt. Vettore and Laga Mts. faults. Ann. Geophys..

[21] Tavarnelli, E. *et al*. Implications of fault reactivation and structural inheritance in the Cenozoic tectonic evolution of Italy in *The Geology of**Italy* (eds. Crescenti, U., D’Offizi, S., Merlini, S. & Sacchi,R.) Special Volume, 201–214 (Societa Geologica Italiana, 2004).

[22] Montone, P. & Mariucci, M. T. The new release of the Italian contemporary stress map. *Geophys. J. Int*. **205**, 1525–1531; gji/ggw100, (2016).

[23] Mariucci, M. T. & Montone, P. *IPSI 1.3, Database of Italian Present-day Stress Indicators*. Istituto Nazionale di Geofisica e Vulcanologia (INGV), 10.6092/INGV.IT-IPSI.1.3 (2019).

[24] Chiaraluce L (2017). The 2016 Central Italy seismic sequence: a first look at the mainshocks, aftershocks, and source models. Seismol. Res. Lett..

[25] Pizzi A, Di Domenica A, Gallovic F, Luzi L (2017). & Puglia, R. Fault segmentation as constraint to the occurrence of the main shocks of the 2016 Central Italy seismic sequence. Tectonics.

[26] Chiarabba C, De Gori P, Cattaneo M, Spallarossa D, Segou M (2018). Faults geometry and the role of fluids in the 2016-2017 Central Italy seismic sequence. Geophys. Res. Lett..

[27] Civico R (2018). Surface ruptures following the 30 October 2016 Mw 6.5 Norcia earthquake, central Italy. J. Maps.

[28] Caporali A (2018). A quantitative approach to the loading rate of seismogenic sources in Italy. Geophys. J. Int..

[29] Roselli P, Marzocchi W, Mariucci MT, Montone P (2018). Earthquake Focal Mechanism Forecasting in Italy for PSHA purposes. Geophys. J. Int..

[30] Lavecchia G (2016). Ground deformation and source geometry of the 24 August, 2016 Amatrice earthquake (Central Italy) investigated through analytical numerical modeling of DInSAR measurements and structural-geological data. Geophys. Res. Lett..

[31] Falcucci E (2018). The Campotosto seismic gap in between the 2009 and 2016-2017 seismic sequence of central Italy and the role of inherited lithospheric faults in regional seismotectonic settings. Tectonics.

[32] Scognamiglio L (2018). Complex fault geometry and rupture dynamics of the Mw 6.5 2016 October 30th central Italy earthquake. J. Geophys. Res..

[33] Walters RJ (2018). Dual control of fault intersections on stop-start rupture in the 2016 Central Italy seismic sequence. Earth & Planet. Sci. Lett..

[34] Bonini L (2019). Testing different tectonic models for the source of the Mw 6.5, 30 October 2016, Norcia earthquake (central Italy): a youthful normal fault, or negative inversion of an old thrust?. Tectonics.

[35] Cheloni D, Falcucci E, Gori S (2019). Half-graben rupture geometry of the 30 October 2016 Mw 6.6 Mt. Vettore-Mt. Bove earthquake, central Italy. J. Geophys. Res..

[36] Mancinelli P (2019). Gravity and magnetic modeling of Central Italy: insights into the depth extent of the seismogenic layer. Geochem. Geophys. Geosyst..

[37] EMERGEO W. G. Coseismic effects of the 2016 Amatrice seismic sequence: first geological results. *Ann. Geophys*. **59**(5), 10.4401/ag-7195 (2016).

[38] Villani F (2018). A database of the coseismic effects following the 30 October 2016 Norcia earthquake in Central Italy. Scientific Data.

[39] Brozzetti F (2019). High-resolution field mapping and analysis of the August - October 2016 coseismic surface faulting (central Italy earthquakes): slip distribution, parameterization, and comparison with global earthquakes. Tectonics.

[40] Galli P (2019). The awakening of the dormant Mount Vettore fault (2016 central Italy earthquake, Mw 6.6): paleoseismic clues on its millennial silences. Tectonics.

[41] Gardner GHF, Gardner LW, Gregory AR (1974). Formation velocity and density: the diagnostic basics for stratigraphic traps. Geophysics.

[42] Scrocca, D. *et al*. RETRACE-3D (centRal italy EarThquakes integRAted Crustal model) Project: preliminary results. *Congresso SGI-SIMP 2018*, Società Geologica Italiana, Roma p. 323 (2018).

[43] Bally A, Burbi L, Cooper C, Ghelardoni R (1986). Balanced sections and seismic reflection profiles across the central Apennines. Mem. Soc. Geol. Ital..

[44] Barchi M (1998). The structural style of the Umbria-Marche fold and thrust belt. Mem. Soc. Geol. Ital..

[45] Scarascia S, Cassinis R, Federici F (1998). Gravity modelling of deep structures in the Northern-Central Apennines. Mem. Soc. Geol. It..

[46] Pauselli C, Federico C (2003). Elastic modeling of the Alto Tiberina normal fault (central Italy): geometry and lithological stratification influences on the local stress field. Tectonophysics.

[47] Mirabella F, Barchi M, Lupattelli A, Stucchi E, Ciaccio MG (2008). Insights on the seismogenic layer thickness from the upper crust structure of the Umbria-Marche Apennines (central Italy). Tectonics.

[48] Mirabella F, Brozzetti F, Lupattelli A, Barchi MR (2011). Tectonic evolution of a low-angle extensional fault system from restored cross sections in the Northern Apennines (Italy). Tectonics.

[49] Barchi MR, Alvarez W, Shimabukuro DH (2012). The Umbria-Marche Apennines as a double orogen: observations and hypotheses. Ital. J. Geosci..

[50] Porreca M (2018). Seismic reflection profiles and subsurface geology of the area interested by the 2016–2017 earthquake sequence (Central Italy). Tectonics.

[51] Bigi S, Casero P, Ciotoli G (2011). Seismic interpretation of the Laga basin; constraints on the structural setting and kinematics of the Central Apennines. J. Geol. Soc. London.

[52] Trippetta F, Collettini C, Barchi MR, Lupattelli A, Mirabella F (2013). A multidisciplinary study of a natural example of a CO2 geological reservoir in central Italy. Int. J. Greenh. Gas Con..

[53] Guerri M, Cammarano F, Connolly JAD (2015). Effects of chemical composition, water and temperature on physical properties of continental crust. Geochem. Geophys. Geosyst..

[54] Diaferia G., Cammarano F. (2017). Seismic Signature of the Continental Crust: What Thermodynamics Says. An Example From the Italian Peninsula. Tectonics.

[55] Barberi, F. & Scandone, P. (eds) Structural Model of Italy, scale 1:500.000. *CNR Progetto Finalizzato Geodinamica* (1983).

[56] Mariotti G, Doglioni C (2000). The dip of the foreland monocline in the Alps and Apennines. Earth & Planet. Sci. Lett..

[57] Di Stefano R (2011). Fault zone properties affecting the rupture evolution of the 2009 (Mw 6.1) L’Aquila earthquake (central Italy): insights from seismic tomography. Geophys. Res. Lett..

[58] Matrullo E, De Matteis R, Satriano C, Amoroso O, Zollo A (2013). An improved 1-D seismic velocity model for seismological stu dies in the Campania–Lucania region (Southern Italy). Geophys. J. Int..

[59] Brocher TM (2005). Empirical relations between elastic wavespeeds and density in the earth’s crust. B. Seismol. Soc. Am..

[60] Ludwig, W. J., Nafe, J. E. & Drake, C. L. Seismic refraction in *The**Sea* (ed. Maxwell A. E.), Vol. 4, 53–84 (Wiley-Interscience 1970).

[61] Burlini L, Tancredi S (1998). Experimental study of the seismic properties of Isola d’Elba metamorphic basement (Tuscany, Italy). Mem. Soc. Geol. Ital..

[62] Ponziani F (1995). Crustal shortening and duplication of the Moho in the northern Apennines: a view from seismic refraction data. Tectonophysics.

[63] De Franco R (1998). Dss-war experiment in support of the Crop03 project. Mem. Soc. Geol. Ital..

