# Supplementary material for: Constraints on the Structure of the Shallow Crust in Central Italy from Geophysical Log Data
Source: Sci Rep. 2020 Mar 2;10:3834. doi: 10.1038/s41598-020-60855-0 (PMC7051982; doi:10.1038/s41598-020-60855-0)
Supplement: Supplementary file 1 — Supplementary information. [file 41598_2020_60855_MOESM1_ESM.pdf]

Supplementary information

Constraints on the Structure of the Shallow Crust in Central Italy from Geophysical Log Data

Paola Montone* and Maria Teresa Mariucci

Istituto Nazionale di Geofisica e Vulcanologia, Sezione Roma1
Via di Vigna Murata 605 – 00143 Roma
Italy

*paola.montone@ingv.it

WELL number	LAT N	LON E	Depth Interval		Data quality * (A the best)	Sonic transit-time (µs/feet)	Litho-stratigraphic unit
			Depth-max (m)	Depth-min (m)			
1	43,555	12,016	1740	1260	A	65	EO-MIO
1	43,555	12,016	1830	1740	A	60	EO-MIO
1	43,555	12,016	2030	1830	A	55	C-EO
1	43,555	12,016	2175	2030	A	50	J-C
1	43,555	12,016	2315	2175	A	50	J
1	43,555	12,016	2400	2315	A	50	TR
1	43,555	12,016	2480	2400	A	48	TR
1	43,555	12,016	2600	2480	A	50	J
1	43,555	12,016	2690	2600	A	55	J-C
1	43,555	12,016	2818	2690	A	50	J-C
1	43,555	12,016	3058	2818	A	50	TR
1	43,555	12,016	3120	3058	A	50	J
1	43,555	12,016	3180	3120	B	60	undefined
1	43,555	12,016	3250	3180	B	65	undefined
1	43,555	12,016	3295	3250	A	65	undefined
1	43,555	12,016	3660	3295	A	50	TR
1	43,555	12,016	3858	3660	A	48	TR
1	43,555	12,016	4000	3858	A	60	A
1	43,555	12,016	4140	4000	A	55	A
1	43,555	12,016	4250	4140	A	48	TR
1	43,555	12,016	4300	4250	A	65	A
1	43,555	12,016	4390	4300	A	60	A
1	43,555	12,016	4485	4390	A	50	A
1	43,555	12,016	4659	4485	A	55	A
1	43,555	12,016	4720	4659	A	58	A
2	43,381	12,42	248	9	C	75	FMA
2	43,381	12,42	775	248	A	75	FMA
2	43,381	12,42	905	775	B	70	FMA
2	43,381	12,42	1010	905	B	65	FMA
2	43,381	12,42	1076	1010	A	70	FMA
2	43,381	12,42	1139	1076	B	75	EO-MIO
2	43,381	12,42	1180	1139	A	60	C-EO
2	43,381	12,42	1436	1180	B	55	C-EO
2	43,381	12,42	1540	1436	B	55	J-C
2	43,381	12,42	1750	1540	A	50	J-C
2	43,381	12,42	1885	1750	A	55	J-C
2	43,381	12,42	1950	1885	A	55	J-C
2	43,381	12,42	2220	1950	A	50	J-C
2	43,381	12,42	2400	2220	A	50	J
2	43,381	12,42	2525	2400	A	55	J
2	43,381	12,42	2625	2525	B	60	J
2	43,381	12,42	2687	2625	A	55	J
2	43,381	12,42	2840	2687	B	55	TR
2	43,381	12,42	4775	2840	A	50	TR
2	43,381	12,42	5070	4775	B	50	TR
2	43,381	12,42	5600	5070	A	45	TR

3	43,232	12,283	140	8,1	C	90	FMA
3	43,232	12,283	326	140	A	85	FMA
3	43,232	12,283	810	326	A	50	TR
3	43,232	12,283	1300	810	A	48	TR
3	43,232	12,283	1410	1300	A	45	TR
3	43,232	12,283	1480	1410	A	50	TR
3	43,232	12,283	2000	1480	A	46	TR
3	43,232	12,283	2790	2000	A	48	TR
3	43,232	12,283	3030	2790	A	55	TR
3	43,232	12,283	3120	3030	A	60	V
3	43,232	12,283	3200	3120	A	65	V
3	43,232	12,283	3480	3200	A	60	V
3	43,232	12,283	3750	3480	A	65	V
3	43,232	12,283	3800	3750	A	60	V
3	43,232	12,283	3850	3800	A	65	V
3	43,232	12,283	3900	3850	A	60	V
3	43,232	12,283	3970	3900	A	65	V
3	43,232	12,283	3990	3970	A	60	V
3	43,232	12,283	4051	3990	A	65	V
4	43,179	13,317	130	7,8	D	110	MIO-PL
4	43,179	13,317	300	130	B	110	MIO-PL
4	43,179	13,317	450	300	B	95	MIO-PL
4	43,179	13,317	535	450	B	90	MIO-PL
4	43,179	13,317	625	535	B	85	MIO-PL
4	43,179	13,317	870	625	B	95	MIO-PL
4	43,179	13,317	1085	870	A	90	MIO-PL
4	43,179	13,317	1200	1085	A	85	MIO-PL
4	43,179	13,317	1255	1200	A	80	MIO-PL
4	43,179	13,317	1295	1255	A	75	MIO-PL
4	43,179	13,317	1326	1295	B	60	MIO-PL
4	43,179	13,317	1525	1326	A	80	MIO-PL
4	43,179	13,317	1575	1525	A	85	MIO-PL
4	43,179	13,317	1700	1575	A	75	MIO-PL
4	43,179	13,317	1796	1700	A	70	MIO-PL
4	43,179	13,317	1833	1796	A	77	EO-MIO
4	43,179	13,317	1871	1833	A	70	EO-MIO
4	43,179	13,317	1920	1871	A	60	EO-MIO
4	43,179	13,317	1980	1920	A	70	EO-MIO
4	43,179	13,317	2025	1980	A	65	EO-MIO
4	43,179	13,317	2155	2025	A	70	EO-MIO
4	43,179	13,317	2245	2155	B	70	EO-MIO
4	43,179	13,317	2343	2245	A	70	EO-MIO
4	43,179	13,317	2386	2343	A	60	C-EO
4	43,179	13,317	2721	2386	A	55	C-EO
4	43,179	13,317	2762	2721	A	60	J-C
4	43,179	13,317	2950	2762	A	50	J-C
4	43,179	13,317	3050	2950	A	55	J
4	43,179	13,317	3200	3050	A	50	J
5	42,597	13,219	200	9,2	D	70	MIO-PL
5	42,597	13,219	1175	200	B	70	MIO-PL

5	42,597	13,219	1196	1175	B	80	MIO-PL
5	42,597	13,219	1235	1196	A	70	EO-MIO
5	42,597	13,219	1315	1235	A	75	EO-MIO
5	42,597	13,219	1385	1315	A	65	EO-MIO
5	42,597	13,219	1400	1385	A	80	EO-MIO
5	42,597	13,219	1410	1400	A	65	EO-MIO
5	42,597	13,219	1500	1410	A	70	EO-MIO
5	42,597	13,219	1615	1500	A	65	EO-MIO
5	42,597	13,219	1630	1615	A	55	EO-MIO
5	42,597	13,219	1715	1630	A	65	EO-MIO
5	42,597	13,219	1793	1715	A	60	EO-MIO
5	42,597	13,219	1950	1793	A	55	EO-MIO
5	42,597	13,219	2000	1950	A	50	EO-MIO
5	42,597	13,219	2100	2000	A	60	EO-MIO
5	42,597	13,219	2152	2100	A	55	EO-MIO
5	42,597	13,219	2215	2152	A	65	EO-MIO
5	42,597	13,219	2260	2215	A	60	EO-MIO
5	42,597	13,219	2274	2260	A	55	EO-MIO
5	42,597	13,219	2841	2274	A	50	C-EO
5	42,597	13,219	2853	2841	A	60	J-C
5	42,597	13,219	3225	2853	A	50	J-C
5	42,597	13,219	3275	3225	A	55	J-C
5	42,597	13,219	3310	3275	A	50	J-C
5	42,597	13,219	3335	3310	A	55	J-C
5	42,597	13,219	3418	3335	A	49	J-C
5	42,597	13,219	3645	3418	A	48	J
5	42,597	13,219	4135	3645	A	47	J
5	42,597	13,219	4375	4135	A	48	J
5	42,597	13,219	4515	4375	A	46	J
5	42,597	13,219	4805	4515	A	48	J
5	42,597	13,219	5015	4805	B	45	TR
5	42,597	13,219	5105	5015	A	48	TR
5	42,597	13,219	5195	5105	B	48	TR
5	42,597	13,219	5245	5195	A	45	TR
5	42,597	13,219	5265	5245	B	50	TR
5	42,597	13,219	5350	5265	A	45	TR
5	42,597	13,219	5395	5350	A	48	TR
5	42,597	13,219	5440	5395	B	50	TR
5	42,597	13,219	5500	5440	A	45	TR
5	42,597	13,219	5520	5500	B	60	TR
5	42,597	13,219	5550	5520	B	75	TR
5	42,597	13,219	5580	5550	A	50	TR
5	42,597	13,219	5660	5580	A	45	TR
5	42,597	13,219	5740	5660	A	50	TR
5	42,597	13,219	5766	5740	A	48	TR
6	42,541	13,335	225	8,5	D	75	MIO-PL
6	42,541	13,335	425	225	B	75	MIO-PL
6	42,541	13,335	525	425	B	65	MIO-PL
6	42,541	13,335	620	525	B	90	MIO-PL
6	42,541	13,335	875	620	B	80	MIO-PL

6	42,541	13,335	1000	875	A	70	MIO-PL
6	42,541	13,335	1150	1000	B	65	MIO-PL
6	42,541	13,335	1200	1150	A	60	MIO-PL
6	42,541	13,335	1390	1200	B	60	EO-MIO
6	42,541	13,335	1525	1390	A	50	EO-MIO
6	42,541	13,335	1775	1525	A	60	EO-MIO
6	42,541	13,335	2225	1775	A	55	C-EO
6	42,541	13,335	2541	2225	A	52	C-EO
7	42,648	13,697	189	5	C	85	PL
7	42,648	13,697	380	189	A	85	PL
7	42,648	13,697	770	380	A	80	PL
7	42,648	13,697	820	770	B	70	PL
7	42,648	13,697	950	820	B	80	PL
7	42,648	13,697	1140	950	A	80	PL
7	42,648	13,697	1260	1140	A	75	PL
7	42,648	13,697	1620	1260	A	70	PL
7	42,648	13,697	1650	1620	A	80	PL
7	42,648	13,697	2030	1650	A	70	PL
7	42,648	13,697	2290	2030	A	65	PL
7	42,648	13,697	2430	2290	A	70	MIO-PL
7	42,648	13,697	2870	2430	A	65	MIO-PL
7	42,648	13,697	2990	2870	A	60	MIO-PL
7	42,648	13,697	3219	2990	A	65	MIO-PL
8	42,549	13,803	570	7,8	C	110	PL
8	42,549	13,803	660	570	A	90	PL
8	42,549	13,803	700	660	B	115	PL
8	42,549	13,803	970	700	B	90	PL
8	42,549	13,803	1070	970	A	95	PL
8	42,549	13,803	1240	1070	B	80	PL
8	42,549	13,803	1400	1240	B	85	PL
8	42,549	13,803	1620	1400	B	75	PL
8	42,549	13,803	1985	1620	A	80	PL
9	42,545	13,868	200	7,4	C	105	PL
9	42,545	13,868	355	200	A	105	PL
9	42,545	13,868	465	355	A	100	PL
9	42,545	13,868	600	465	A	95	PL
9	42,545	13,868	1250	600	A	90	PL
9	42,545	13,868	1642	1250	B	80	PL
10	42,529	13,865	200	6,2	C	100	PL
10	42,529	13,865	450	200	A	100	PL
10	42,529	13,865	590	450	A	95	PL
10	42,529	13,865	990	590	A	90	PL
10	42,529	13,865	1380	990	A	85	PL
10	42,529	13,865	1620	1380	B	80	PL
10	42,529	13,865	1980	1620	B	75	PL
11	42,5	13,881	560	10,2	C	90	PL
11	42,5	13,881	670	560	A	95	PL
11	42,5	13,881	780	670	B	90	PL
11	42,5	13,881	945	780	A	90	PL
11	42,5	13,881	1030	945	B	80	PL

11	42,5	13,881	1780	1030	B	85	PL
11	42,5	13,881	2110	1780	B	75	PL
11	42,5	13,881	2430	2110	B	90	PL
11	42,5	13,881	2522	2430	A	85	PL
11	42,5	13,881	2890	2520	B	80	MIO-PL
11	42,5	13,881	3066	2890	A	70	EO-MIO
11	42,5	13,881	3206	3066	A	60	EO-MIO
11	42,5	13,881	3650	3206	A	52	C-EO
11	42,5	13,881	3710	3650	B	60	J-C
11	42,5	13,881	3940	3710	A	50	J-C
11	42,5	13,881	3997	3940	A	60	J-C
11	42,5	13,881	4105	3997	A	50	J-C
11	42,5	13,881	4520	4105	A	48	J
11	42,5	13,881	4540	4520	B	70	J
11	42,5	13,881	5100	4540	A	48	J
11	42,5	13,881	5440	5100	A	46	J
11	42,5	13,881	5470	5440	B	60	J
11	42,5	13,881	6440	5470	A	46	J
11	42,5	13,881	6820	6440	A	45	TR
12	42,416	13,855	250	4,7	D	100	PL
12	42,416	13,855	400	250	B	100	PL
12	42,416	13,855	530	400	A	80	PL
12	42,416	13,855	765	530	A	85	PL
12	42,416	13,855	875	765	A	80	PL
12	42,416	13,855	930	875	A	85	PL
12	42,416	13,855	1000	930	B	80	PL
12	42,416	13,855	1135	1000	B	75	PL
12	42,416	13,855	1175	1135	A	80	PL
12	42,416	13,855	1205	1175	A	70	PL
12	42,416	13,855	1335	1205	A	75	PL
12	42,416	13,855	1385	1335	A	70	PL
12	42,416	13,855	1580	1385	B	85	PL
12	42,416	13,855	1700	1580	A	85	MIO-PL
12	42,416	13,855	1725	1700	A	70	MIO-PL
12	42,416	13,855	1980	1725	A	80	MIO-PL
12	42,416	13,855	2000	1980	B	80	MIO-PL
12	42,416	13,855	2225	2000	A	80	MIO-PL
12	42,416	13,855	2420	2225	A	75	MIO-PL
12	42,416	13,855	2575	2420	A	80	MIO-PL
12	42,416	13,855	2660	2575	A	75	MIO-PL
12	42,416	13,855	2802	2660	A	80	MIO-PL
12	42,416	13,855	2980	2802	A	70	EO-MIO
12	42,416	13,855	3102	2980	A	75	EO-MIO
12	42,416	13,855	3295	3102	A	70	EO-MIO
12	42,416	13,855	3395	3295	A	55	EO-MIO
12	42,416	13,855	3442	3395	A	60	EO-MIO
12	42,416	13,855	3586	3442	A	53	C-EO
13	42,421	13,915	198	12,9	C	105	PL
13	42,421	13,915	330	198	A	105	PL
13	42,421	13,915	570	330	A	95	PL

13	42,421	13,915	1040	570	A	90	PL
13	42,421	13,915	1310	1040	A	85	PL
13	42,421	13,915	1730	1310	B	80	PL
13	42,421	13,915	1810	1730	A	70	PL
13	42,421	13,915	2035	1810	B	80	PL
13	42,421	13,915	2140	2035	A	80	PL
13	42,421	13,915	2370	2140	A	70	EO-MIO
13	42,421	13,915	2500	2370	A	60	EO-MIO
13	42,421	13,915	2595	2500	A	53	C-EO
13	42,421	13,915	2665	2595	A	65	J-C
13	42,421	13,915	2862	2665	A	50	J-C
13	42,421	13,915	3346	2862	A	50	J
14	42,421	13,915	205	7,7	D	100	PL
14	42,421	13,915	330	205	A	100	PL
14	42,421	13,915	455	330	A	95	PL
14	42,421	13,915	775	455	A	90	PL
14	42,421	13,915	900	775	A	85	PL
14	42,421	13,915	1270	900	A	90	PL
14	42,421	13,915	1415	1270	B	80	PL
14	42,421	13,915	1500	1415	B	85	PL
14	42,421	13,915	1760	1500	B	75	PL
14	42,421	13,915	1850	1760	A	70	PL
14	42,421	13,915	1960	1850	B	75	PL
14	42,421	13,915	2230	1960	B	80	PL
14	42,421	13,915	2400	2230	A	70	EO-MIO
14	42,421	13,915	2469	2400	A	55	EO-MIO
14	42,421	13,915	2563	2469	A	50	C-EO
15	42,843	13,912	900	8,1	C	115	PL
15	42,843	13,912	1150	900	A	115	PL
15	42,843	13,912	1275	1150	A	110	PL
15	42,843	13,912	1360	1275	A	105	PL
15	42,843	13,912	1405	1360	A	110	PL
15	42,843	13,912	1525	1405	A	105	PL
15	42,843	13,912	1580	1525	A	100	PL
15	42,843	13,912	1750	1580	A	105	PL
15	42,843	13,912	1855	1750	A	100	PL
15	42,843	13,912	1880	1855	A	95	PL
15	42,843	13,912	2040	1880	A	100	PL
15	42,843	13,912	2100	2040	A	95	PL
15	42,843	13,912	2275	2100	A	90	PL
15	42,843	13,912	2375	2275	A	95	PL
15	42,843	13,912	2390	2375	A	100	PL
15	42,843	13,912	2525	2390	A	95	PL
15	42,843	13,912	2600	2525	A	90	PL
15	42,843	13,912	2915	2600	A	95	PL
15	42,843	13,912	3050	2915	B	80	PL
15	42,843	13,912	3075	3050	A	90	PL
15	42,843	13,912	3090	3075	B	80	PL
15	42,843	13,912	3125	3090	B	90	PL
15	42,843	13,912	3140	3125	A	85	PL

16	42,784	13,797	250	7,6	C	125	PL
16	42,784	13,797	332	250	A	125	PL
16	42,784	13,797	383	332	B	100	PL
16	42,784	13,797	460	383	A	120	PL
16	42,784	13,797	670	460	A	115	PL
16	42,784	13,797	750	670	A	110	PL
16	42,784	13,797	820	750	A	115	PL
16	42,784	13,797	930	820	A	120	PL
16	42,784	13,797	960	930	A	115	PL
16	42,784	13,797	1170	960	A	105	PL
16	42,784	13,797	1280	1170	A	100	PL
16	42,784	13,797	1550	1280	A	95	PL
16	42,784	13,797	1680	1550	A	90	PL
16	42,784	13,797	1760	1680	A	95	PL
16	42,784	13,797	1860	1760	A	100	PL
16	42,784	13,797	2060	1860	A	95	PL
16	42,784	13,797	2500	2060	A	90	PL
16	42,784	13,797	2700	2500	A	85	PL
16	42,784	13,797	3001	2700	A	95	PL
17	42,792	13,808	730	9	D	140	PL
17	42,792	13,808	1520	730	D	125	PL
17	42,792	13,808	2235	1520	C	85	PL
17	42,792	13,808	2420	2235	A	85	PL
17	42,792	13,808	2465	2420	B	85	PL
17	42,792	13,808	2495	2465	B	95	PL
17	42,792	13,808	2685	2495	A	85	PL
17	42,792	13,808	2780	2685	B	75	PL
17	42,792	13,808	2875	2780	A	75	PL
17	42,792	13,808	3285	2875	A	70	PL
17	42,792	13,808	3305	3285	A	80	PL
17	42,792	13,808	3345	3305	A	65	PL
17	42,792	13,808	3375	3345	A	75	PL
17	42,792	13,808	3450	3375	A	70	PL
17	42,792	13,808	3658	3450	A	65	PL
17	42,792	13,808	3765	3658	A	60	PL
17	42,792	13,808	3860	3765	A	70	PL
17	42,792	13,808	3965	3860	A	65	PL
17	42,792	13,808	4070	3965	A	70	PL
17	42,792	13,808	4150	4070	A	65	PL
17	42,792	13,808	4195	4150	A	70	PL
17	42,792	13,808	4275	4195	A	65	PL
17	42,792	13,808	4415	4275	A	60	PL
17	42,792	13,808	4559	4415	A	65	PL
17	42,792	13,808	4650	4559	B	80	PL
17	42,792	13,808	4775	4650	A	75	PL
17	42,792	13,808	4905	4775	A	65	PL
17	42,792	13,808	4940	4905	A	75	PL
17	42,792	13,808	5043	4940	B	75	PL
18	42,693	13,853	340	5,8	B	100	PL
18	42,693	13,853	440	340	A	120	PL

18	42,693	13,853	480	440	B	75	PL
18	42,693	13,853	662	480	B	90	PL
18	42,693	13,853	890	662	A	110	PL
18	42,693	13,853	1070	890	A	105	PL
18	42,693	13,853	1150	1070	A	95	PL
18	42,693	13,853	1490	1150	A	100	PL
18	42,693	13,853	1800	1490	A	95	PL
18	42,693	13,853	2130	1800	A	90	PL
18	42,693	13,853	2600	2130	A	85	PL
18	42,693	13,853	2720	2600	A	75	PL
18	42,693	13,853	3290	2720	A	70	PL
18	42,693	13,853	3360	3290	A	65	PL
19	42,673	13,858	165	4	C	130	PL
19	42,673	13,858	250	165	A	130	PL
19	42,673	13,858	330	250	A	125	PL
19	42,673	13,858	425	330	A	120	PL
19	42,673	13,858	475	425	A	115	PL
19	42,673	13,858	575	475	A	110	PL
19	42,673	13,858	650	575	A	115	PL
19	42,673	13,858	725	650	A	113	PL
19	42,673	13,858	780	725	A	110	PL
19	42,673	13,858	855	780	A	105	PL
19	42,673	13,858	910	855	A	110	PL
19	42,673	13,858	1040	910	A	105	PL
19	42,673	13,858	1100	1040	A	100	PL
19	42,673	13,858	1125	1100	A	105	PL
19	42,673	13,858	1250	1125	B	95	PL
19	42,673	13,858	1360	1250	A	100	PL
19	42,673	13,858	1400	1360	A	92	PL
19	42,673	13,858	1450	1400	A	100	PL
19	42,673	13,858	1750	1450	A	95	PL
19	42,673	13,858	1836	1750	A	90	PL
20	42,605	13,984	250	5,6	D	135	Q
20	42,605	13,984	310	250	A	135	Q
20	42,605	13,984	350	310	A	140	Q
20	42,605	13,984	540	350	A	130	Q
20	42,605	13,984	710	540	A	120	PL
20	42,605	13,984	925	710	A	115	PL
20	42,605	13,984	1050	925	A	110	PL
20	42,605	13,984	1175	1050	A	105	PL
20	42,605	13,984	1450	1175	A	100	PL
20	42,605	13,984	1675	1450	A	95	PL
20	42,605	13,984	1925	1675	A	90	PL
20	42,605	13,984	2050	1925	A	95	PL
20	42,605	13,984	2125	2050	A	90	PL
20	42,605	13,984	2465	2125	A	100	PL
20	42,605	13,984	2575	2465	A	95	PL
20	42,605	13,984	3525	2575	A	90	PL
20	42,605	13,984	3735	3525	A	85	PL
21	42,557	13,905	300	5,2	D	130	PL

21	42,557	13,905	425	300	A	130	PL
21	42,557	13,905	480	425	A	125	PL
21	42,557	13,905	530	480	A	120	PL
21	42,557	13,905	625	530	A	115	PL
21	42,557	13,905	715	625	A	110	PL
21	42,557	13,905	885	715	A	100	PL
21	42,557	13,905	1100	885	A	95	PL
21	42,557	13,905	1325	1100	A	90	PL
21	42,557	13,905	1550	1325	A	85	PL
21	42,557	13,905	2070	1550	A	80	PL
21	42,557	13,905	2425	2070	A	75	PL
21	42,557	13,905	2510	2425	A	70	PL
21	42,557	13,905	2610	2510	A	75	PL

* Sonic Data Quality: A, high; B, low; C, inferred data; D, assumed data
